# Tenascin-W is a better cancer biomarker than tenascin-C for most human solid tumors

**DOI:** 10.1186/1472-6890-12-14

**Published:** 2012-09-04

**Authors:** Florence Brellier, Enrico Martina, Martin Degen, Nathalie Heuzé-Vourc’h, Agnès Petit, Thomas Kryza, Yves Courty, Luigi Terracciano, Christian Ruiz, Ruth Chiquet-Ehrismann

**Affiliations:** 1 Friedrich Miescher Institute for Biomedical Research, Novartis Research Foundation, Basel, Switzerland; 2 Faculty of Sciences, University of Basel, Basel, Switzerland; 3 Université François Rabelais, EA 6305, F-37032, Tours, France; 4 Centre d'Etude des Pathologies Respiratoires, UMR 1100/EA6305, F-37032, Tours, France; 5 Institute of Pathology, University Hospital Basel, Basel, Switzerland; 6 Present address: Department of Dermatology, Brigham and Women’s Hospital, Harvard Skin Disease Research Center, Harvard Medical School, Boston, MA, USA

## Abstract

**Background:**

Tenascins are large glycoproteins found in the extracellular matrix of many embryonic and adult tissues. Tenascin-C is a well-studied biomarker known for its high overexpression in the stroma of most solid cancers. Tenascin-W, the least studied member of the family, is highly expressed in the stroma of colon and breast tumors and in gliomas, but not in the corresponding normal tissues. Other solid tumors have not been analyzed. The present study was undertaken to determine whether tenascin-W could serve as a cancer-specific extracellular matrix protein in a broad range of solid tumors.

**Methods:**

We analyzed the expression of tenascin-W and tenascin-C by immunoblotting and by immunohistochemistry on multiple frozen tissue microarrays of carcinomas of the pancreas, kidney and lung as well as melanomas and compared them to healthy tissues.

**Results:**

From all healthy adult organs tested, only liver and spleen showed detectable levels of tenascin-W, suggesting that tenascin-W is absent from most human adult organs under normal, non-pathological conditions. In contrast, tenascin-W was detectable in the majority of melanomas and their metastases, as well as in pancreas, kidney, and lung carcinomas. Comparing lung tumor samples and matching control tissues for each patient revealed a clear overexpression of tenascin-W in tumor tissues. Although the number of samples examined is too small to draw statistically significant conclusions, there seems to be a tendency for increased tenascin-W expression in higher grade tumors. Interestingly, in most tumor types, tenascin-W is also expressed in close proximity to blood vessels, as shown by CD31 co-staining of the samples.

**Conclusions:**

The present study extends the tumor biomarker potential of tenascin-W to a broad range of solid tumors and shows its accessibility from the blood stream for potential therapeutic strategies.

## Background

During recent years increasing evidence has emerged showing that the microenvironment plays a prominent role in determining tumor behavior [1-4]. Tumor progression is influenced and controlled by activation of nearby stromal cells, including fibroblasts, endothelial cells and macrophages. In their activated states, these cells modulate and reorganize the extracellular matrix and create a congenial microenvironment for the tumor cells. Accepting an active role of the microenvironment for tumor progression, it will be important to consider how the activated tumor stroma can be harnessed for clinical benefit. Extracellular matrix proteins specifically expressed in the tumor stroma could thus represent promising predictive/diagnostic biomarkers or target molecules for therapeutics.

Tenascin-C (TNC) and tenascin-W (TNW) are two members of the tenascin family of large extracellular matrix glycoproteins (see [5] for information on the structure of tenascins). Their functions are associated with cellular mechanisms such as adhesion modulation, motility, proliferation and differentiation [5]. Both proteins share highest expression during embryonic development and reduced and very restricted expression in adult tissues [6]. TNC, the best-described family member, reappears during pathological conditions such as cancer, inflammation or wound-healing [7,8]. Soon after its initial identification, TNC was proposed as a stromal marker in breast cancer [9]. Since then, many more studies shown that TNC expression could have predictive value for local tumor recurrence and metastatic dissemination in many aggressive cancers (for reviews see [2,10,11]. Its cancer-specific expression has been exploited to make TNC a promising target for different anti-cancer therapies, including the delivery of cytokines or radionuclides to the tumor using TNC-specific monoclonal antibodies [12-14] or aptamers [15,16].

Similar physiological expression patterns as well as shared functions of TNW with TNC prompted us to investigate the presence of the newest tenascin family member, TNW in different human cancers. TNW expression was detected in a large majority of human breast tumors, showing enrichment in low-grade compared to high-grade tumors [17]. In colorectal cancer, TNW expression was restricted to the tumor-associated microenvironment, while the protein was not detectable in the corresponding healthy tissue [18]. In brain tumors, all glioma subtypes tested (oligodendroglioma, astrocytoma and glioblastoma) were enriched in TNW in comparison to healthy control brain tissues [19]. Noticeably, in all glioblastoma samples analyzed the localization of TNW was perivascular [19].

Encouraged by the highly tumor-specific expression of TNW in breast, colon, and brain tumors, we now extended our expression study to many more types of solid tumors. We show here, that TNW expression displays an even higher specificity for cancer-related microenvironments than TNC. Hence, TNW represents a novel attractive cancer biomarker of broad potential for tumor detection, prediction and targeting approaches.

## Methods

### Tissue samples

Protein extracts from uterus, spleen, liver, lung, cerebrum and heart control tissues were from autoptic material obtained from the Institute of Pathology from the University Hospital of Basel, Switzerland. Pancreas, kidney and colon control whole tissue homogenates were purchased from BioCat GmbH (Heidelberg, Germany). Protein extracts from breast, pancreas and kidney tumors were obtained from the Institute of Pathology of the University Hospital of Basel, Switzerland. All extracts were prepared as described before [17].

Melanomas, kidney and lung tumors used for immunohistochemistry were part of frozen tissue microarrays (TMA) obtained from the Institute of Pathology of the University Hospital of Basel, Switzerland. References for their construction can be found elsewhere [17]. The melanoma TMA was constructed from frozen tissue samples of 34 malignant melanomas (5 primary tumors, 8 metastases from different organs and 21 lymph node metastases) and 6 control healthy cutaneous tissue samples. The kidney TMA was constructed from frozen tissue samples of 84 kidney tumors (70 clear cell renal carcinomas, 6 papillary renal carcinomas, 6 chromophobe renal cell carcinomas and 2 oncocytoma) and 10 healthy control kidney tissues. The lung TMA was constructed from frozen tissue samples of 74 lung tumors (40 squamous carcinomas, 19 adenocarcinomas and 15 undifferentiated carcinomas). Additional lung tumor samples were obtained from the University Hospital of Tours, France. The latter collection contained frozen tissues from matched samples of tumor and adjacent non-tumor tissue. They were obtained from 21 patients who had undergone lung cancer resection as their primary therapy without preoperative radiation or chemotherapy. These non-small cell lung cancers consisted of 10 adenocarcinomas, 10 squamous carcinomas and 1 undifferentiated carcinoma. The non-malignant tissue samples were taken from sites at least 3 cm away from the edge of the tumor. The histological diagnosis was determined by two pathologists.

Studies were performed in compliance with the Declaration of Helsinki and in accordance with the guidelines of the ethical committee of the University of Basel, Switzerland or with French bioethical regulations.

### Western blot analysis

Tissue samples were thawed on ice, minced and homogenized in RIPA lysis buffer. After determination of protein concentration by a Bio-Rad Protein assay, 25 μg of protein were separated by SDS-PAGE (6%) and electroblotted to polyvinylidene difluoride membranes. Equal loading and transfer of protein was confirmed by staining the membranes with amidoblack. After a one hour blocking step in 5% milk powder in Tris-buffered saline (TBS), membranes were incubated overnight with the rabbit polyclonal antiserum pAb (3 F/4) raised against human TNW (1:750), the mouse monoclonal antibody B28-13 raised against human TNC (1:100), the V-9131 mouse monoclonal antibody against vinculin (1:2000; Sigma). After incubation for 1 hour with anti-mouse IgG or anti-rabbit IgG coupled to horseradish peroxidase, blots were developed using Super Signal (Pierce) for TNC and TNW and ECL reagent (GE Healthcare) for vinculin followed by exposure to Kodak BioMax MR Films.

### Immunostaining and quantification

Chromogenic and fluorescent detections were performed on 9 μm-thick cryosections using the Discovery XT automated stainer (Ventana Medical Systems) with standard and customized procedures, respectively. Frozen tissue slides were dried for 1 hr at room temperature, fixed for 10 min at 4°C in cold acetone and then introduced into the automate. For chromogenic stainings, slides were first blocked twice for 12 min with the AB Block reagent (Ventana). They were then incubated for 1 hr at 37°C with the mouse monoclonal 56O antibody raised against human TNW (1:1000) [18] or the B28-13 anti-TNC antibody (1:1000). They were then treated for 32 min at 37°C with a biotinylated anti-mouse secondary antibody (1:200; Jackson Immunoresearch laboratories 715-065-150) and developed with DAB Map detection kit (Ventana). Counterstainings were obtained with hematoxylin and bluing reagent (Ventana). For immunofluorescent stainings, slides were incubated for 1 hour at 37°C with anti-TNW mAb 56O (1:50), anti-TNC mAb B28-13 (1:50) or anti-CD31 (1:200; M0823, DAKO). They were then treated for 32 min at 37°C with Alexa Fluor 647 donkey anti-mouse IgG and Alexa Fluor 488 goat anti-rabbit IgG secondary antibodies (1:200; Invitrogen A31571 and A11029, respectively), carefully rinsed by hand and mounted with prolong Gold reagent (Invitrogen). Pictures were either acquired with a Mirax Slidescanner (Zeiss AG, Zurich, Switzerland) using a 20×/0.5 lens (0.2 μm/pixel) and converted into standard TIFF format or with a Nikon Eclipse 80i microscope equipped with a Leica DFC420 color camera. For quantification, the area of the section stained by TNW or TNC was measured using ImageJ by setting the color threshold as follows: hue 0–40, saturation 50–255, luminosity 0–200. The resulting area was divided by the total area of the section to obtain a percentage. The samples were then classified in four categories according to the amount of staining: 0%, no staining; 1%-10%, low; 11%-40%, moderate; >40%, high. In order to compare tumor type or grade with the tenascin stainings a numerical score was assigned to each category (no staining = 0, low = 1, moderate = 2, and high = 3) and the average scores for each tumor type/grade was calculated.

## Results

### TNW is not detectable in most adult organs

**Figure 1:**
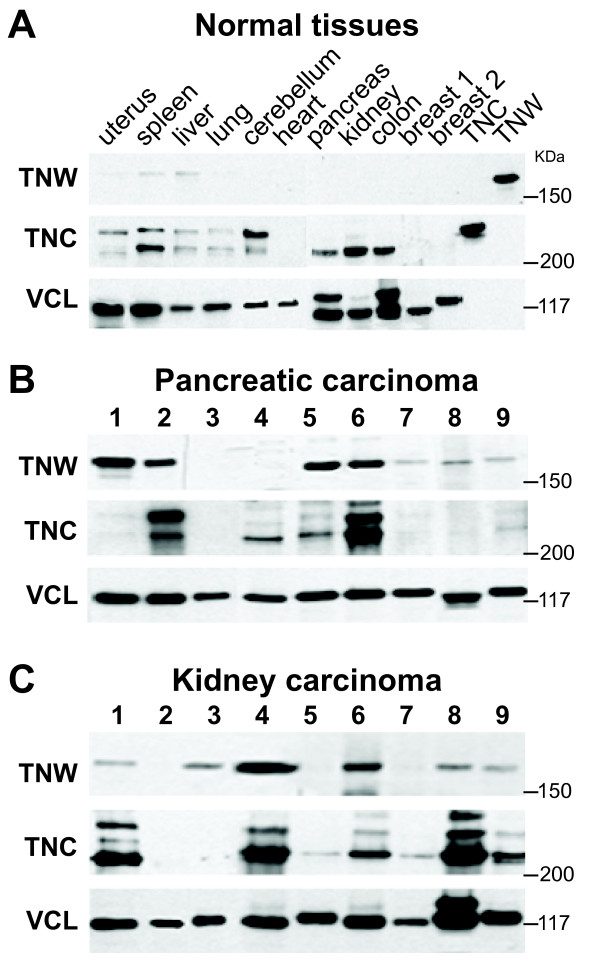
**Immunoblot analysis of TNW and TNC protein expression in various healthy human adult organs (A), in pancreatic carcinomas (B) and in kidney carcinomas (C).** Blots were also probed for vinculin (VCL) to check equal loadings of protein samples. **A**) Note the presence of TNC in all but one organ tested, whereas TNW is undetectable in the majority of organs. The last two lanes contain 25 ng of purified TNC or TNW, respectively. **B**) Extracts from nine pancreatic carcinomas were tested for their TNW and TNC levels. Note that TNW is expressed in most of the pancreatic carcinomas tested. **C**) Extracts of nine kidney carcinomas were tested for their TNW and TNC expression. TNW was observed in most of the kidney carcinomas tested.

To compare TNW and TNC protein expression in healthy adult humans, we analyzed tissue extracts from ten different organs and analyzed their protein content on western-blots (Figure 1A). In spleen and liver extracts very faint bands for TNW were observed. In agreement with our recent observations, we did not detect any TNW in healthy human breast, colon and brain tissues [17-19]. Furthermore, normal uterus, lung, heart, pancreas and kidney appeared devoid of TNW, too. In contrast, TNC was detectable in all organs tested, except for normal breast and heart tissues. As has been described earlier [17-20], we detected different TNC isoforms in our immunoblot analysis of normal human tissues, although in pancreas, kidney, and colon only the lower molecular weight TNC isoform was observed. 

Our earlier studies showed TNW overexpression in human breast, colon, as well as brain tumors [17-19]. To obtain a more complete neoplasia-associated expression profile of TNW, we tested here whether TNW enrichment can also be found in solid tumors originating from other organs.

### TNW is detectable in pancreatic carcinomas

Pancreatic carcinoma extracts from nine different patients were analyzed by western-blotting for their expression of TNW and TNC (Figure 1B). In contrast to healthy pancreas tissue (Figure 1A), most pancreatic carcinomas (7/9) did express detectable amounts of TNW. Among the seven TNW positive tumors, four contained very high levels of TNW, as shown by the high intensity of the corresponding bands (patients # 1, 2, 5, and 6), and three samples displayed moderate levels (patients # 7–9). According to the intensity of the western-blot bands, TNC was observed in most of the samples varying from barely detectable (patients # 1, 7–9) to intermediate (patients # 4, 5) and to high levels (patient # 2, 6). The levels of TNW and TNC did not correlate and in the sample from patient #1 with the highest TNW level TNC was barely detectable (Figure 1B). While in normal pancreas tissue only a low molecular weight isoform of TNC was detectable (Figure 1A), pancreas tumor tissues expressed additional higher molecular weight isoforms (Figure 1B). So far we do not have any evidence for the existence of TNW splice-variants (Figure 1).

### TNW is detectable in kidney tumors

TNW and TNC expression was analyzed by western-blotting in kidney carcinoma samples, and their amounts were estimated by the intensity of the corresponding bands (Figure 1C). TNW was detected in most cases (6/9): 2 out of 9 expressed very high levels of TNW, 4 intermediate levels and in 3 samples TNW was not detectable. TNC was also observed in most samples (7/9), but again could not be correlated with TNW expression.

**Figure 2:**
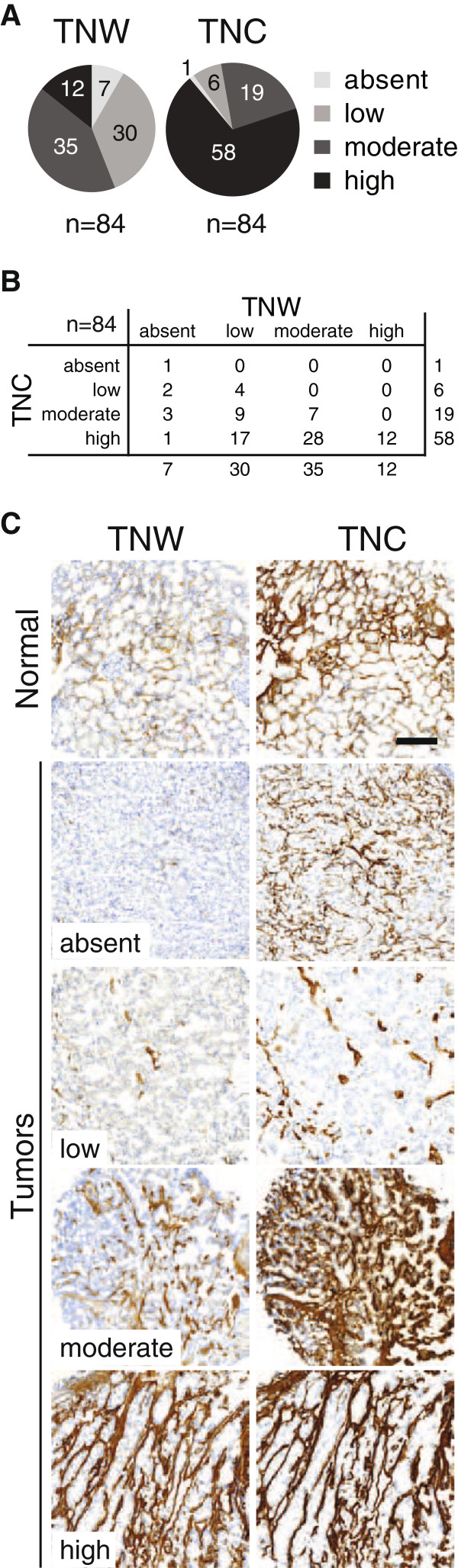
**Immunohistochemical analysis of kidney tumor samples.** Eighty-four kidney tumor samples, spotted on a frozen TMA were stained for expression of TNW and TNC. The area of the section stained for TNW or TNC was quantified and the samples were classified in four categories (absent, low, moderate and high) as described in Materials and Methods. The number of tumors classified in each category for TNW and TNC is shown as pie charts (**A**). A double-entry table details how many tumors show each combination of TNW/TNC expression (**B**). Top panel in C shows normal kidney tissues stained for TNW (left) and TNC (right). Representative pictures of tumors of each category (absent; low; moderate; high) are shown for TNW (C, left panel) with the corresponding tumor area stained for TNC in parallel (C, right panel). Every picture is acquired at the same magnification (Scale bar = 200 μm).

**Figure 3:**
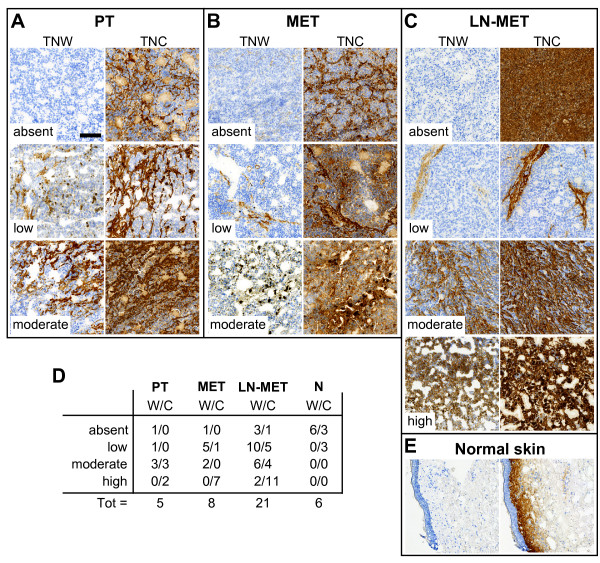
**Immunohistochemical analysis of melanoma samples.** Thirty-four melanoma tissues from a frozen TMA were stained for their expression of TNW and TNC. The area of the section stained for TNW or TNC was quantified and the samples were classified as in Figure 2. Representative pictures of each tumor type are grouped in different panels: **A**) primary tumors (PT), **B**) metastases (MET), **C**) lymph node metastases (LN-MET). From top to bottom, pictures in the left column of each panel represent increasing scores of TNW from absent, low, moderate to high (only in panel C) with the corresponding tumor stained for TNC in the right column of each panel. Panel E shows normal skin tissue stained for TNW (left) and TNC (right). Every picture is acquired at the same magnification (Scale bar = 200 μm). Table D summarizes the distribution of the scores for each tenascin in the different groups.

In parallel, a frozen kidney tumor TMA (n = 84) was stained by immunohistochemistry for expression of TNW and TNC. For each tumor sample, we quantified expression levels of both tenascins and classified them into the following 4 categories with respect to their tenascin expression: absent, low, moderate and high, as described in Materials and Methods (Figure 2A,B). Although we could not detect TNW by western-blot in healthy kidney tissue (Figure 1A), immunohistochemical analysis revealed slight, but clearly visible expression of TNW in most of the control kidney samples while the remaining samples were unstained (see representative image in Figure 2C). As can be seen in the pie chart more than half of the tumors (56%) expressed moderate or high levels of TNW while for TNC this was the case for 92% of all kidney tumors on the TMA (Figure 3A). Interestingly, TNW was never in a higher expression category than TNC (Figure 2B). Using a numerical score for each category (see Materials and methods) the average scores for each tumor type was calculated. The average scores for the levels of TNW and TNC of the 70 clear cell carcinomas were 1.7 ± 0.10 and 2.69 ± 0.07 respectively, for the 6 papillary carcinomas 1.33 ± 0.33 and 2.67 ± 0.33, for the 6 chromophobe carcinomas 1.17 ± 0.31 and 2 ± 0.26 and for the 2 oncocytoma 1 and 1. This suggests that the tenascin levels may correlate with the tumor type. However, more samples would be necessary to strictly prove such a correlation.

### TNW is overexpressed in melanomas

A frozen TMA containing 34 melanoma samples including primary tumors and metastases was stained by immunohistochemistry for TNW and TNC. Each individual tumor staining was quantified and categorized as described above (Figure 3). Representative pictures for each TNW group for each type of sample are shown in parallel to the matching TNC stainings of the same tumor patient. Figure 3A shows stainings of primary tumors (PT), Figure 3B metastases (MET) and Figure 3C lymph node metastases (LN-MET). These tumor samples can be compared to the healthy skin tissue shown in Figure 3E. TNW was not detectable in any normal skin sample whereas TNC was observed in the dermis of three out of six samples (Figure 3D), just below the basement membrane underlying the basal epidermal cell layer, as has been described previously [21]. A summary of all stainings is presented in a table (Figure 3D). It shows that 60% (3 out of 5) of primary melanomas (PT) had moderate or high expression of TNW, while TNC was detectable in all of the primary tumor samples with a staining classified at least as moderate. In samples obtained from metastasis from diverse locations (i.e. spleen, lung, skin), TNW had moderate staining in 25% (2 out of 8) of the samples, while TNC is strongly expressed in 87% (7 out of 8) of samples. Similar proportions were observed in samples of lymph node metastasis: 38% of samples (8 out of 21) showed moderate or high TNW, while 71% (15 out of 21) of the samples displayed at least moderate TNC staining. Similar to the kidney TMA analysis, TNW expression was never found in a higher expression category than TNC. It is noteworthy that the only two samples in which TNW is classified in the highest category of expression are both lymph node metastases.

### TNW is overexpressed in lung tumors

**Figure 4:**
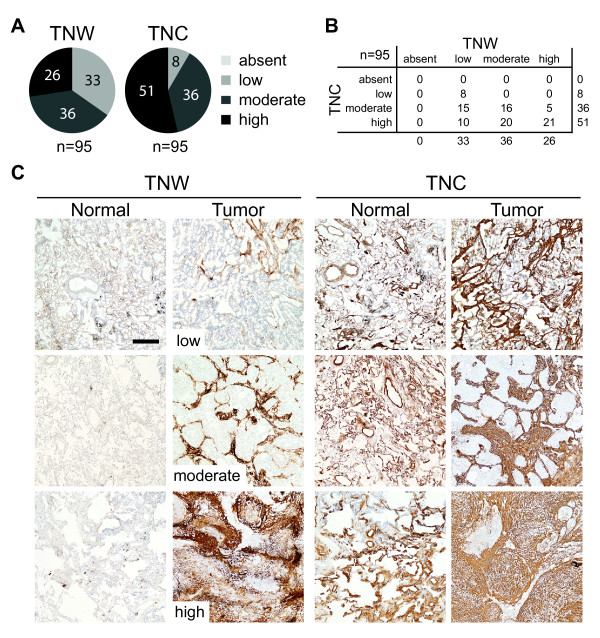
**Immunohistochemical analysis of lung tumor samples.** Ninety-five lung tumor sections were stained for their expression of TNC and TNW and classified as described in Figure 2. The number of tumors classified in each category for TNC and TNW is shown as pie charts (**A**). A double-entry table details how many tumors show each combination of TNW/TNC expression (**B**). Twenty-one pairs of matching lung normal/tumor lung tissue samples were stained for their expression of TNW and TNC. Representative stainings are shown: From top to bottom, tumor pictures represent increasing scores of TNW from low, moderate to high with the corresponding tissues stained for TNC in the right panels. From top to bottom an adenocarcinoma, a squamous carcinoma and an undifferentiated carcinoma are shown (**C**). Every picture is acquired at the same magnification (Scale bar = 200 μm).

In our analysis of lung tumors we stained a total of 95 tumor samples for the presence of TNW and TNC. Immunohistochemical analysis showed that TNW was detectable in all 95 tumor samples (Figure 4). For 21 of these samples we were able to analyze matching adjacent normal lung parenchyma that had been biopsied at least 3 cm away from the edge of the tumor. These corresponding healthy tissues did not display any detectable TNW expression demonstrating that TNW expression was restricted to tumor associated stroma. In contrast, TNC was also present in the normal lung tissue adjacent to the tumors. In many of the lung tumor samples, we could observe a very typical pattern of TNW expression: a fibrous staining (parallel lines forming bundles) surrounding nests of tumor cells (Figure 4C, #9297). This suggests that TNW is mainly expressed by stromal cells surrounding the carcinoma cells. Quantification of the stained area of tumors from an extended cohort of patients (n = 95) revealed that TNW expression was never completely absent in any of the tumors, a feature shared by TNC (Figure 4A,B). 65% of the tumor samples had at least moderate TNW expression while for TNC this was the case for 92% of the samples. With the exception of 5 cases, TNC was in the same or higher expression category as TNW (Figure 4B). However, since TNC expression levels in control lung tissues have been much higher than TNW levels, TNW seems to be much more tumor-specific than TNC in the case of the lung. Information about the stage of the tumor was available for 69 of the 95 samples included in the present study. Interestingly, the average level of TNW seems to correlate with tumor grade: the two grade I tumors both have a TNW score of 1 (TNC score is 2); grade II tumors (n = 19) have an average TNW score of 1.74 ± 0.15 (TNC: 2.47 ± 0.18); tumors with grade intermediate between II and III (n = 6) have an average score of 1.83 ± 0.30 (TNC: 2.33 ± 0.33); grade III tumors (n = 27) have average score of 2.18 ± 0.17 (TNC: 2.55 ± 0.11); finally, the average TNW score of grade IV tumors (n = 15) is 2.07 ± 0.23 (TNC: 2.6 ± 0.16). Although the number of samples examined is too small to draw statistically significant conclusions, there seems to be a tendency for increased TNW expression in higher grade tumors.

### TNW is expressed around blood vessels

**Figure 5:**
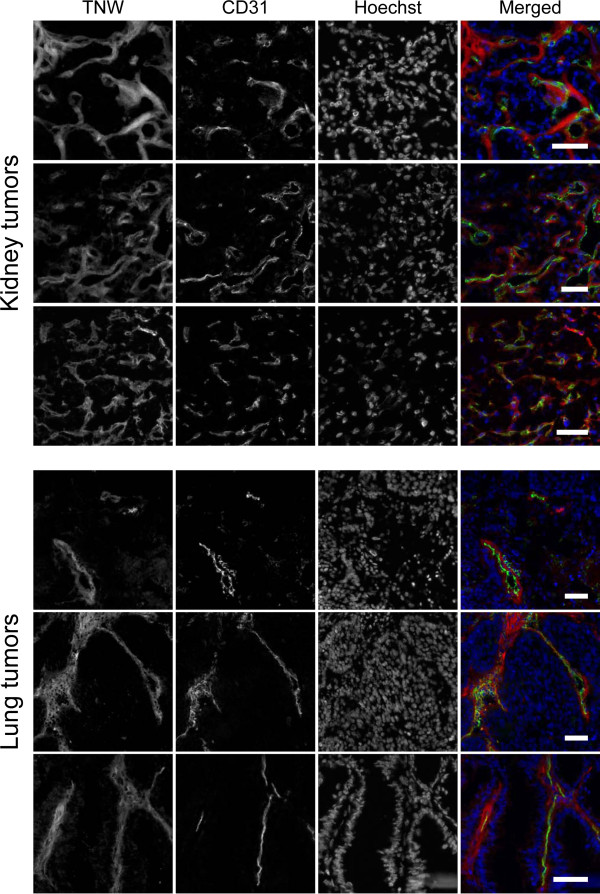
**Double immunofluorescence staining of TNW and CD31 in kidney tumors (upper panel) and lung tumors (lower panels).** Tumor cryosections were used for fluorescent detection of TNW (red), CD31 (green) and DAPI (blue). Each row represents a different tumor sample. Scale bars = 50 μm.

Our previous study on TNW expression in brain tumors showed for the first time that TNW is expressed around blood vessels of gliomas [19]. Here, we investigated whether this observation holds true as well for tumors originating from other organs. For this purpose, we co-stained lung and kidney tumors for TNW and CD-31, an established blood vessel marker (Figure 5). In most of the tumor samples a prominent co-localization between TNW and blood vessels (CD31 positive) was observed. These results confirmed our earlier study in brain tumors and suggest that TNW is often expressed adjacent to blood vessels, independently of vessel size. TNW co-localization with blood vessels was also observed in additional analyses of colon, breast, ovarian and prostate tumors (data not shown). Thus, the presence of TNW around tumor blood vessels seems to be widespread. 

## Discussion

Recent studies on TNW have identified this protein as a candidate biomarker for breast tumors [17], colorectal tumors [18] as well as gliomas [19]. In addition to these tumor sites, we report in the present study that TNW is also prominently expressed in melanomas, pancreas, kidney and lung carcinoma samples. Importantly, we were not able to detect it in the normal corresponding organs, except for a slight presence in normal kidney. The tumor-specific expression was especially striking for the lung for which we had clinical material available to compare 21 tumors with matching healthy tissue fragments of the same patient. All of these 21 tumor/non-tumor pairs revealed a specific tumor-restricted TNW staining, whereas the non-tumor samples were all negative. Therefore, TNW might be a diagnostic marker for lung cancer and its expression seems to increase with tumor grade. TNW was also expressed in the majority of kidney tumors tested. Although western-blot analysis did not reveal the presence of TNW in a kidney control tissue extract, we could observe a slight TNW staining by immunohistochemistry in all 10 control kidney tissues analyzed. This result is in agreement with our previous observation that TNW can be found in the kidney of adult mice [22]. TNW and TNC levels appeared on average to be higher in clear cell carcinomas than in papillary or chromophobe renal cell carcinomas and lowest in oncocytoma, suggesting a correlation with severity of the disease. In addition, we could also observe overexpression of TNW in ovarian cancer samples and in prostate tumors (data not shown). Our general observation that TNW is overexpressed in the stroma surrounding many tumors, but absent in most corresponding control tissues, qualifies TNW as a tumor biomarker. This is less the case for TNC, as TNC overexpression has been correlated with various conditions other than cancers, such as inflammation, infection, asthma, fibrosis and wound healing (for reviews see [7,8]).

So far, TNW overexpression has been exclusively described in the context of tumorigenesis and osteogenesis [6], which suggests that it might be a more specific tumor marker than TNC. However, in the case of TNC certain splice variants with additional fibronectin type 3 domains have been shown to be more tumor-specific than the shortest isoform [23,24]. Therefore, isoforms-specific antibodies against TNC can also be used as tumor-specific markers [24,25]*.* This is also the case for another ubiquitous extracellular matrix protein, namely fibronectin, which was shown to have cancer-specific splice variants which can be used as targets for antibody-mediated therapy in human patients [26,27]. We can not exclude so far a possible association between TNW and other types of tissue remodeling conditions such as inflammation or wound healing. However, we were unable to detect TNW either in biopsies from patients with inflammatory bowel diseases (even in areas where TNC is highly expressed – our unpublished data), nor in wound healing in the mouse [28]. It remains to be seen whether TNW is elevated in sera of patients suffering from solid tumors other than breast and colon, where elevated TNW serum levels have been described [18]. The same might be the case for pancreas, kidney or lung tumor patients as these tumors show strong TNW staining *in situ*.

Here we have found that TNW is expressed in close vicinity of blood vessels in a wide range of tumors including kidney, colon, breast, ovary and prostate tumors. In another study we have found that TNW acts as a pro-angiogenic factor *in vitro*[19]. Therefore, we can speculate that *in vivo* TNW could act on tumor endothelial cells, influencing their migration and thus contributing to the expansion of the endothelial network. What triggers TNW expression in the tumor stroma and more particularly next to the blood vessels remains to be established, but it likely involves complex tumor-microenvironment interactions. Interestingly, a similar deposition outside the endothelial basement membrane has already been described for large TNC splice variants in newly formed blood vessels [23,29]. Use of a renal carcinoma xenograft model has revealed that the source of these perivascular large TNC molecules were the carcinoma cells themselves [29]. It would be of high interest to establish whether carcinoma cells could also be the source of blood vessels associated TNW.

In several tumors, there is now compelling evidence that the capacity for a tumor to grow and proliferate lies in a small fraction of cells, called cancer stem cells (CSC) or cancer initiating cells [30]. Similar to normal stem cells, CSC have the ability to self-renew and to differentiate into a variety of proliferating cells that make up the tumor mass. It can be speculated that maintenance of CSCs depends upon a specific stem cell niche microenvironment that protects CSCs from therapeutics. Therefore, targeting these microenvironments might result in more effective treatments of cancers. Interestingly, TNC has been reported to maintain an ABCB5+ subpopulations of melanoma-initiating cells thereby promoting melanoma progression [31]. Moreover, for several cancers, cancer stem cell populations reside in perivascular niches as their preferred microenvironment [32-35]. Given the tumor-specific overexpression of TNW and its perivascular staining pattern, one can speculate about a role for TNW in maintaining CSC’s and offering them a congenial niche.

## Conclusions

The present study extends the potential of TNW as cancer biomarker to a large variety of solid tumors. Given both its specific expression in tumors compared to corresponding healthy tissues and its proximity to tumor blood vessels (making it accessible *via* the bloodstream) we propose TNW as a good candidate for selective delivery of anticancer medicine. The perivascular deposition pattern of TNW however will limit this strategy to antibody-drug conjugates able to cross the blood vessel walls.

## Competing interests

Some of the presented results have been included in the patent applications WO2003/080663, WO2009/053354 and WO2010/119021.

## Authors’ contributions

FB performed immunostainings of kidney, lung and melanoma TMAs and drafted the manuscript. EM performed the immunofluorescence stainings, quantified all immunostainings, performed the statistical analyses and made the figures. MD performed the immunoblots and contributed to the writing. NHV, AP, TK, YC collected, classified and stained the lung cancer tissues and the normal control tissues of the same patients. CR and LT provided the kidney, lung and melanoma frozen TMAs and classified the tumor and patient data. RCE conceived of the study and participated in its design and coordination and helped to write the manuscript. All authors read and approved the final manuscript.

## Pre-publication history

The pre-publication history for this paper can be accessed here:

http://www.biomedcentral.com/1472-6890/12/14/prepub
